# Prehospital Intubation in Patients with Isolated Severe Traumatic Brain Injury: A 4-Year Observational Study

**DOI:** 10.1155/2014/135986

**Published:** 2014-01-16

**Authors:** Mazin Tuma, Ayman El-Menyar, Husham Abdelrahman, Hassan Al-Thani, Ahmad Zarour, Ashok Parchani, Sherwan Khoshnaw, Ruben Peralta, Rifat Latifi

**Affiliations:** ^1^Department of Surgery, Section of Trauma Surgery, Hamad General Hospital (HGH), P.O. Box 3050, Doha, Qatar; ^2^Clinical Research, Section of Trauma Surgery, HGH, P.O. Box 3050, Doha, Qatar; ^3^Clinical Medicine, Weill Cornell Medical School, P.O. Box 24144, Doha, Qatar; ^4^Department of Surgery, University of Arizona, P.O. Box 245005, Tucson, AZ, USA

## Abstract

*Objectives*. To study the effect of prehospital intubation (PHI) on survival of patients with isolated severe traumatic brain injury (ISTBI). *Method*. Retrospective analyses of all intubated patients with ISTBI between 2008 and 2011 were studied. Comparison was made between those who were intubated in the PHI versus in the trauma resuscitation unit (TRU). *Results*. Among 1665 TBI patients, 160 met the inclusion criteria (105 underwent PHI, and 55 patients were intubated in TRU). PHI group was younger in age and had lower median scene motor GCS (*P* = 0.001). Ventilator days and hospital length of stay (*P* = 0.01 and 0.006, resp.) were higher in TRUI group. Mean ISS, length of stay, initial blood pressure, pneumonia, and ARDS were comparable among the two groups. Mortality rate was higher in the PHI group (54% versus 31%, *P* = 0.005). On multivariate regression analysis, scene motor GCS (OR 0.55; 95% CI 0.41–0.73) was an independent predictor for mortality. *Conclusion*. PHI did not offer survival benefit in our group of patients with ISTBI based on the head AIS and the scene motor GCS. However, more studies are warranted to prove this finding and identify patients who may benefit from this intervention.

## 1. Introduction

Prehospital intubation (PHI) is a standard approach for early critical care management among severe trauma patients. In particular, establishment of definitive airway is an integral part in the optimal care and management of severe traumatic brain injury (STBI) patients [1]. Several data showed that early prevention of hypoxia at the scene has favorable effect on the survival in patients with STBI [2–5]. An earlier study demonstrated that PHI in isolated STBI patients significantly reduced the mortality from 50% to 23%, with an absolute survival benefit of 27% [6]. However, other data found PHI in head injury patients to be associated with worse outcomes [7–10], even with the use of Rapid Sequence Intubation (RSI) [11]. Many factors have been postulated to be responsible for such adverse outcome. These factors include higher risk of aspiration pneumonia, the effect of laryngoscopy on raising the intracranial pressure, the deleterious effect of hyperventilation and hypocapnia, and the potentially harmful effect of supra normal oxygen tension (hyperoxia) on the injured brain [12–15]. Helm et al. [16], in a prospective study, evaluated the effect of quality of controlled ventilation and airway protection in head injury patients. The authors did not find PHI effective for maintaining optimal oxygenation and ventilation in these patients. Despite high incidence of head injuries, the impact of PHI has not been extensively studied in our region. The aim of the present study is to evaluate the effect of prehospital intubation on the outcome of isolated STBI patients in Qatar.

## 2. Methods

### 2.1. Type of the Study

A retrospective cohort study over period of 4 years from January 2008 to December 2011 was conducted.

### 2.2. Setting

Hamad General Hospital (HGH) is the only tertiary health care center, with a dedicated state of the art Trauma Center and Emergency Medical Services (EMS) equipped with ground and air ambulance services that covers a population of about 1.7 million in Qatar. Our EMS service is following up-to-date evidence-based standard treatment protocols and is staffed by critical care paramedics as well as Emergency Medical Technicians (EMT). Pre-hospital intubation is conducted exclusively by well-trained critical care paramedics (CCP). Qatar Emergency Medical Services has developed educational modules for training CCP in collaboration with external training agencies. A CCP program was established to provide additional clinical skills and expertise and support the improvement of clinical practice throughout HMC Ambulance Service. All CCPs were trained at the HMC-EMS. The training program includes RSI, surgical airway, needle thoracentesis, diagnostic and therapeutic emergency cardiac procedures, the application of a comprehensive critical care medication formulary, and the management of critical care patients during transfer.

Data were extracted from computer database registry. The registry records included demographic data, as well as scene vital signs, Glasgow Coma Score (GCS), motor component of GCS (coded between 1 and 6) [17], and intubation data. In addition, the modes of transportation, mechanism of injury, admission vital signs, head AIS score, injury severity score (ISS), and outcome were also collected. All trauma patients were managed according to advanced trauma life support (ATLS) principles and trauma brain foundation guidelines [18] as appropriate.

### 2.3. Inclusion Criteria

All adult patients of age >14 years are admitted to HGH who required intubation based on GCS and head AIS. Patients with isolated STBI were identified as head Abbreviated Injury Score (AIS) ≥3 with no other regions of AIS >3 and field GCS score ≤8 [7].

All patients were divided into two groups according to the place of intubation: group 1 (intubated at the scene; PHI) and group 2 (intubated in the resuscitation room; TRUI).

### 2.4. Exclusion Criteria

We excluded patients who died within 24 hours after injury (in whom the cause of death may be due to life-threatening hemorrhage or unclear cause), patients who transferred from other hospitals, or those who were intubated in the operating room or intensive care unit.

### 2.5. Primary End Point

All-cause mortality is within 30 days after admission.

### 2.6. Ethical Approval

This study was approved by the Medical Research Center (IRB no. 12134/12) at Hamad Medical Corporation, Qatar.

### 2.7. Statistical Analysis

Data were presented as proportions or mean ± standard deviation (SD) as appropriate. Baseline demographic characteristics, presentation, management, and outcomes were compared between the two groups using the Student's *t*-test for continuous variables and Pearson chi-square (*χ* ^2^) test for categorical variables. Multivariate logistic regression was used to calculate the odds ratio for prediction of mortality. A significant difference was considered when the *P* value was less than 0.05. Data analysis was carried out using the Statistical Package for Social Sciences version 18 (SPSS Inc., Chicago, USA).

## 3. Results

During the study period, there were 1665 patients admitted with diagnosis of traumatic brain injury. Only 160 patients had ISTBI (defined by GCS score ≤8 and head AIS score ≥3) and intubation was carried on for all. 105 (66%) were intubated in the prehospital settings (PHI group) and 55 (34%) were intubated in the trauma resuscitation room (TRUI group) (Table 1). All cases were intubated orally using RSI. Most patients were males with mean age of 31 ± 14 years. Traffic related injuries were the predominant mechanism of injury. Overall median motor component of GCS was 1(1–6) and the overall mortality rate was 46% (Table 1).

Patients in PHI group were younger in age and had significantly lower median scene motor GCS [1(1–5) versus 3(1–5); *P* = 0.001]. Majority of patients in TRUI group were transported to the hospital by ground EMS (98% versus 76%; *P* = 0.001) (Table 1), whereas helicopter was used to transport more patients in PHI group (24% versus 2%; *P* = 0.001). Further, oxygen saturation, systolic blood pressure, and pulse rates were comparable among the two groups. More patients in TRUI group underwent craniotomy (25% versus 6%; *P* = 0.001). Ventilator days [5(1–29) versus 3(1–19); *P* = 0.014] and hospital length of stay [22(1–410) versus 9(1–380); *P* = 0.006] were significantly higher in TRUI group. The mean ISS, ICU length of stay, initial blood pressure, blood alcohol levels, and complication rate (pneumonia and ARDS) were comparable among the two groups (Table 1). The mortality rate was significantly higher in the PHI group (54% versus 31%, *P* = 0.005) (Table 1). On multivariate regression analysis after adjusting for covariates (age, ISS, motor GCS, place of intubation, and EMS time), scene motor GCS (OR 0.55; 95%CI 0.41–0.73) was found to be an independent predictor for mortality (Table 2).

## 4. Discussion

This study demonstrates that the overall mortality rate in severe traumatic brain injury patients is about 46% based on the head AIS ≥3 and GCS ≤8. This finding is consistent with other academic centers that follow brain trauma foundation guidelines in the management of severe traumatic brain injury [23]. In the current study, low motor component of GCS was an independent predictor of mortality in intubated ISTBI patients regardless of the place of intubation.  Table 3 shows a review of literature for prehospital intubation in severe traumatic head injury patients [6, 7, 9, 19–22]. Prehospital intubation of severely injured patients who cannot maintain adequate airway is common practice worldwide. Such practice, however, has not improved the outcomes in injured patients. Moreover, PHI was found to be deleterious in many studies [7–12]. Consistent with these studies, our data showed around twofold increased rate of mortality in PHI group compared to TRUI group. Murray et al. [7] also showed that patients with STBI requiring PHI had an increased mortality (81%) when compared with non-intubated patients (43%). The possible explanation for higher mortality rate is that majority of the patient in PHI group had higher incidence of penetrating injury and had higher head AIS and mean ISS. In addition, early death (within 48 hours) was not excluded, as those usually constitute the most lethal injuries.

Bochicchio et al. [9] studied prospectively 191 patients with STBI, after exclusion of those who died within the first 48 hours. Although, there were no significant differences in GCS, head AIS, and ISS between the two groups, PHI had a significantly greater mortality compared with patients intubated in the emergency department. PHI patients also were more likely to have died from complications such as respiratory failure. However, our study showed no significant difference in rates of pneumonia and ARDS between the two groups.

On the other hand, survival benefit was demonstrated in a study conducted by Winchell and Hoyt [6]. PHI was associated with dramatic improved survival in patients with isolated STBI (from 50% to 23%), and that effect was more pronounced after exclusion of lethal injuries with GCS of 3. However, in that study, the analysis did not adjust for potentially important factors such as hypotension or hypoxia to validate the hypothesis that prevention of secondary insult was attributed to this positive outcome.

Davis et al. [22] studied the impact of prehospital intubation on outcome using large database of head injured patients. The authors concluded that patients with head AIS ≥4 and/or GCS of 3–8 had a higher mortality rate, if they were intubated in pre-hospital setting compared to emergency department intubation (63% versus 36%, *P* = 0.001).

Although the present study was not intended to examine the effects of mode of transportation on patient's outcome, most head injured patients were transferred by ground transportation, while the percentage of pre-hospital intubation was significantly higher in the air flights. This was attributed to many factors discussed in previous studies, such as severity of injury, training of paramedics, and time factor during transfer [24]. Recent studies have demonstrated improved safety and effectiveness with doctor delivered PHI compared to the paramedical staff [25–27]. However, in Qatar, highly trained critical care paramedics are delivering prehospital critical care services. Our analysis addresses the controversial issue of PHI in head injury cases and this may pave the way for further improvement of EMS services.

*Limitations*. The limitations of this study include its retrospective design, as it was based on administrative data that lacks many points to capture, such as number of attempted or failed intubations, duration of the intubation attempt, and use of medications. No disability data are available to determine the long-term functional outcome of survivors. Possibility of survival bias is present in favor of in-hospital intubation as the patient population being compared is not truly identical. We tried to avoid the later by excluding early mortality cases, as they constitute the most lethal injuries.

## 5. Conclusion

Prehospital intubation continues to be a controversial issue. In isolated STBI based on AIS, PHI is associated with (but not cause of) a worse outcome in comparison to in-hospital intubation. PHI may be a marker for high risk in selected trauma population. So, more studies are needed for in-depth analysis for the lack of benefit of prehospital in this group of patients.

## Figures and Tables

**Table 1:** Demographics of all intubated isolated severe traumatic brain injury cases.

	All intubated* *n* = 160	Prehospital intubation *n* = 105 (65%)	TRU intubation *n* = 55 (34%)	*P *value
Age (yrs; mean ± SD)	31 ± 14	30 ± 14	34 ± 15	0.14
Males (%)	96.5	95	98	0.35
Nationality				
Qatari (%)	22	20	23	0.27 for all
Non-Arab (%)	78	80	77	
Mechanism of injury				0.92 for all
Fall from height (%)	12	13.5	11	
Traffic related injuries (%)	81	79	83	
Fall of heavy object (%)	3	3	2	
EMS time (mean ± SD)	63 ± 34	70 ± 30	52 ± 33	0.07
Mode of transport				
Ground EMS (%)	84	76	98	0.001 for all
Air flight (%)	16	24	2	
ISS (mean ± SD)	27 ± 10	28 ± 8	27 ± 10	0.32
Scene data				
Glasgow motor score (median)	1 (1–6)	1 (1–5)	3 (1–5)	0.001
O_2_ saturation	92 ± 10	91 ± 11	94 ± 8	0.16
SBP (mean ± SD)	133 ± 32	129 ± 29	142 ± 40	0.05
Pulse (mean ± SD)	93 ± 30	93 ± 28	92 ± 30	0.78
CT head (%)	91.5	89	100	0.009
Craniotomy (%)	14	6	25	0.001
Ventilator days (median)	3 (1–29)	3 (1–19)	5 (1–29)	0.014
Hospital days (median)	12 (1–410)	9 (1–380)	22 (1–410)	0.006
ICU LOS (mean ± SD)	1 (1-2)	1 (1-2)	1 (1-2)	0.865
Pneumonia (%)	12	11.4	18.2	0.23
ARDS (%)	1.5	1.0	1.8	0.64
Blood alcohol level (median)	44 (7–93)	38 (27–64)	43 (7–66)	0.926
ED disposition				0.11 for all
Admitted to ICU (%)	81	85	86	
Shifted to OR (%)	11	7	13	
Mortality (%)	46	54	31	0.005

*Patients had head AIS ≥ 3 and GCS ≤ 8.

**Table 2:** Multivariate analysis for predictors of mortality among all intubated ISTBI cases.

Variables	*P* value	OR	95% CI	
Craniotomy (Yes versus No)	0.864	0.907	0.297	2.774
Scene motor GCS (reduced versus not)	0.000	0.550	0.414	0.731
Mode of transport*	0.338	0.630	0.245	1.620
Intubation location**	0.157	0.552	0.242	1.256

ISTBI: isolated severe traumatic brain injury; *ground versus air; **PHI versus hospital.

**Table 3:** Review of literature for prehospital intubation studies in severe TBI patients.

Authors	Definition criteria	Patient number	Overall mortality	Outcome
Murray et al. 2000 [7]	Severe TBI (field GCS < 8 and head AIS > 3)	894 TBI: isolated TBI (570)	47.2%	Prehospital intubation failed to demonstrate survival benefits in patients with severe TBI.
Bochicchio et al. 2003 [9]	Severe TBI (GCS score ≤ 8 and a HAIS score ≥ 3). Patients who died within 48 hours of admission were excluded	191 TBI: isolated TBI (68)	16.8%	PHI had significantly increased mortality (23% versus 12.4%, *P* = 0.05). Also, the risk of mortality was 1.85 times higher in PHI group compared to ED intubated patients.
Wang et al. 2004 [19]	Severe TBI (head/neck AIS ≥ 3)	4098	37%	PHI is associated with increased risk of mortality (OR 3.99; 95% CI 3.21 to 4.93) and poor neurologic outcome (OR 1.61; 95% CI 1.15 to 2.26).
Vandromme et al. 2011 [20]	Severe TBI (prehospital GCS score ≤ 8)	149	46.9%	PH intubation is associated with severe TBI but had no increased risk for mortality over ED intubation.
Warner et al. 2007 [21]	Severe TBI (head AIS score > 3), isolated TBI (head AIS score > 3 but no other AIS score > 2).	187 TBI: isolated TBI (95)	24.4%	Targeted PHI is associated with lower mortality after severe TBI.
Winchell and Hoyt 1997 [6]	Head AIS ≥ 4 and GCS ≤ 8	671 TBI: isolated TBI (351)	57%	Field intubation reduced mortality from 57% to 36% in patients with severe TBI and from 50% to 23% in isolated TBI
Davis et al. 2005 [22]	Moderate to severe TBI (head/neck AIS score of ≥ 3).	13,625	22.9%	PHI is associated with increased mortality (55% versus 15%) in comparison to nonintubated patients with moderate to severe TBI.
Present study	Field GCS ≤ 8 and head AIS ≥ 3. Patients who died within first 24 hours were excluded	160 isolated severe TBI	46%	No added benefit in PHI group. Scene motor GCS (OR 0.55; 95% CI 0.41–0.73) had a significant association with mortality.

PHI: Prehospital intubations; TBI: traumatic brain injuries; GCS: Glasgow Coma Scale score; AIS: Abbreviated Injury Scale; ED: emergency department.
